# Contradictory mRNA and protein misexpression of EEF1A1 in ductal breast carcinoma due to cell cycle regulation and cellular stress

**DOI:** 10.1038/s41598-018-32272-x

**Published:** 2018-09-17

**Authors:** Cheng-Yu Lin, Alexandra Beattie, Behzad Baradaran, Eloise Dray, Pascal H. G. Duijf

**Affiliations:** 1 University of Queensland Diamantina Institute, The University of Queensland, Translational Research Institute, 37 Kent Street, Brisbane, QLD 4102 Australia; 2 Immunology Research Center, Tabriz University of Medical Sciences, Tabriz, Iran; 3 Department of Immunology, Faculty of Medicine, Tabriz University of Medical Sciences, Tabriz, Iran; 4 Institute of Health and Biomedical Innovation, Queensland University of Technology, Translational Research Institute, 37 Kent Street, Brisbane, QLD 4102 Australia; 5 Mater Research Institute-The University of Queensland, Translational Research Institute, 37 Kent Street, Brisbane, QLD 4102 Australia

## Abstract

Encoded by *EEF1A1*, the eukaryotic translation elongation factor eEF1α1 strongly promotes the heat shock response, which protects cancer cells from proteotoxic stress, following for instance oxidative stress, hypoxia or aneuploidy. Unexpectedly, therefore, we find that EEF1A1 mRNA levels are reduced in virtually all breast cancers, in particular in ductal carcinomas. Univariate and multivariate analyses indicate that EEF1A1 mRNA underexpression independently predicts poor patient prognosis for estrogen receptor-positive (ER+) cancers. EEF1A1 mRNA levels are lowest in the most invasive, lymph node-positive, advanced stage and postmenopausal tumors. In sharp contrast, immunohistochemistry on 100 ductal breast carcinomas revealed that at the protein level eEF1α1 is ubiquitously overexpressed, especially in ER+ , progesterone receptor-positive and lymph node-negative tumors. Explaining this paradox, we find that EEF1A1 mRNA levels in breast carcinomas are low due to *EEF1A1* allelic copy number loss, found in 27% of tumors, and cell cycle-specific expression, because mRNA levels are high in G1 and low in proliferating cells. This also links estrogen-induced cell proliferation to clinical observations. In contrast, high eEF1α1 protein levels protect tumor cells from stress-induced cell death. These observations suggest that, by obviating *EEF1A1* transcription, cancer cells can rapidly induce the heat shock response following proteotoxic stress, and survive.

## Introduction

Breast cancer is the most common cancer in women. With 1.7 million new cases diagnosed per year, it accounts for about 25% of all cancer diagnoses worldwide^1^. However, only a few prognostic and predictive biomarkers are routinely used for the selection of patients benefitting from specific therapies^2^. Examples include the estrogen receptor (ER) and progesterone receptor (PR) for endocrine therapies and human epidermal growth factor receptor type 2 (HER2) for targeted therapy^3,4^. However, clinical outcomes remain variable^2^. Therefore, new prognostic and predictive breast cancer biomarkers are required for more accurate prognoses and therapeutic decision making.

Chromosome instability (CIN) is a hallmark of cancer and refers to an increased rate of chromosomal abnormalities. Aberrant chromosomal rearrangements, including aneuploidy, partial chromosome gains or losses and translocations, often promote tumorigenesis. They do so by generating fusion genes or gene copy number alterations, which induce unbalanced expression of oncogenic proteins or tumor suppressors^5–7^. Global chromosomal imbalances, such as tetraploidy, may also promote subclonal heterogeneity, and this is strongly associated with poor patient prognosis and drug-resistance^8,9^. Thus, CIN signatures are developed and extensively used as clinical biomarkers^10–12^.

Widespread copy number changes as a result of CIN cause genetic and proteomic imbalances. Work from the Amon laboratory in particular showed that this disrupts proteome homeostasis, affecting protein degradation and folding, and causes proteotoxic stress^13–15^. A so-called ‘heat shock response’ (HSR) is elicited to counteract this cellular stress^16,17^. The HSR is an evolutionarily conserved protective mechanism for cells under intrinsic or environmental stress. It maintains proteome homeostasis by dramatically increasing the production of heat shock proteins (HSPs) in a short period of time to prevent protein misfolding, aggregation, aberrant trafficking and degradation. Recent studies have reported enhanced HSP expression in a broad range of human cancers^18,19^. HSPs can either stabilize oncogenic proteins, such as mutant TP53^18^, or suppress cell death, thus promoting tumor development^20^. HSP expression is regulated by transcription factors named heat shock factors (HSFs), which bind to Heat Shock Elements (HSEs) in promoters via the conserved HSF DNA-binding domain^21^. Heat Shock Factor 1 (HSF1) is the well-established master regulator of HSP expression in mammalian cells and has been shown to support oncogenesis^22^.

Two homologs of the eukaryotic translation elongation factor eEF1α exist: eEF1α1 and eEF1α2. They have well-defined roles in recruiting aminoacyl-tRNAs to the A site of ribosomes during protein synthesis, but only eEF1α1 is ubiquitously expressed. Besides this canonical function, eEF1α1 participates in signal transduction, cell proliferation, cell cycle regulation, apoptosis and the HSR. EEF1α1 overexpression has been observed in hepatocellular carcinoma (HCC) and knock-down reduces HCC cell proliferation and blocks cell cycle progression^23,24^. However, the clinical significance of eEF1α1 in breast cancer has not yet been established.

Here, we study EEF1A1 mRNA and eEF1α1 protein expression in breast cancer and investigate associations between misexpression and clinical parameters. We find that at the mRNA level, EEF1A1 is underexpressed and this is an independent marker for poor patient prognosis in ER+ breast cancer. Reduced mRNA expression is caused by *EEF1A1* copy number loss and cell cycle-associated expression. Strikingly, however, at the protein level eEF1α1 is overexpressed in breast cancer tissues and this protects breast cancer cells from cell death under stress conditions.

## Results

### EEF1A1 mRNA is underexpressed in breast cancers

**Figure 1:**
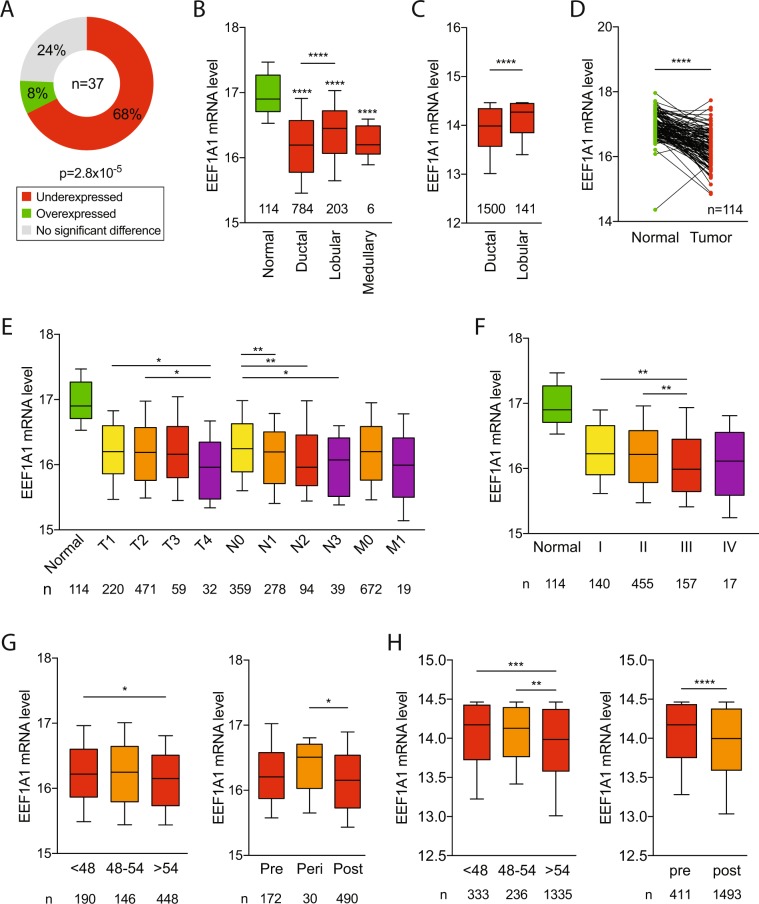
EEF1A1 mRNA is underexpressed in breast cancer. (**A**) EEF1A1 mRNA levels in normal breast and breast cancer were pairwise compared in 37 microarray studies. The graph shows the distribution of studies reporting underexpression or overexpression in tumors or significant differential expression, as indicated. P-value: Chi-square test. (**B**) Box plot of normalized EEF1A1 mRNA expression in indicated breast carcinomas compared to normal breast tissue using the TCGA breast cancer RNAseq dataset. Whiskers show 10–90 percentiles. P-values: Mann-Whitney *U* tests. (**C**) Box plot of normalized EEF1A1 mRNA expression in breast ductal carcinoma compared to lobular carcinoma using the METABRIC dataset. Whiskers show 10–90 percentiles. P-values: Mann-Whitney *U* test. (**D**) Normalized EEF1A1 mRNA expression in matched breast carcinoma and normal breast tissue pairs using the TCGA RNAseq dataset. P-value: Wilcoxon matched-pairs signed rank test. (**E**) Box plot of normalized EEF1A1 mRNA expression in breast tumors for TNM stages, which includes tumor invasion (T1-T4), nodal status (N0-N3) and metastatic states (M0-M1), as indicated. Whiskers show 10–90 percentiles. P-values: Mann-Whitney *U* tests. (**F**) Box plot showing normalized EEF1A1 mRNA expression in breast carcinomas for indicated tumor stages. Whiskers show 10–90 percentiles. P-values: Mann-Whitney *U* tests. (**G**) Box plot showing normalized EEF1A1 mRNA expression in breast tumors for age and menopausal status using the TCGA RNAseq dataset. Whiskers show 10–90 percentiles. P-values: Mann-Whitney *U* tests. (**H**) Box plot showing normalized EEF1A1 mRNA expression in breast tumors for age and menopausal status using the METABRIC dataset. Whiskers show 10–90 percentiles. P-values: Mann-Whitney *U* test. *p < 0.05; **p < 0.01; ***p < 0.001; ****p < 0.0001.

Using 37 analyses from 11 previously reported microarray datasets (Supplementary Table S1), we studied EEF1A1 mRNA expression levels in a broad range of breast cancers, including ductal, lobular, medullary and mucinous breast cancers^25^. Twenty-five out of 37 (68%) showed underexpressed EEF1A1 mRNA levels in breast cancers compared to normal breast tissue. Only 3 datasets (8%) showed overexpressed EEF1A1 mRNA levels in breast cancers (*p* = 2.8 × 10^−5^, Chi-square test; Fig. 1A). Notably, one of these three reports studied benign neoplasms and another involved phyllodes tumors, which may be benign or malignant, indicating that EEF1A1 mRNA is typically underexpressed in malignant breast cancer.

Using data from The Cancer Genomic Atlas (TCGA), we also assessed EEF1A1 mRNA expression levels from an RNAseq platform^26^. We compared 1097 breast tumors to 114 normal breast tissues. Similar to our previous observations, this showed that breast carcinomas – irrespective of their subtype – have significantly decreased mRNA expression compared to normal breast tissue (*p* < 0.0001, Mann-Whitney *U* test; Fig. 1B). Also, comparison between breast tumor histological subtypes showed that EEF1A1 expression is significantly lower in ductal breast carcinoma than in lobular breast carcinoma (*p* < 0.0001, Mann-Whitney *U* test; Fig. 1B). This observation was also confirmed in an independent dataset from METABRIC (*p* < 0.0001; Fig. 1C)^27,28^. In addition, paired analysis demonstrated that EEF1A1 levels are significantly lower in breast tumors compared to their matched normal breast tissues (*p* < 0.0001; Wilcoxon matched-pairs signed rank test, Fig. 1D). Thus, together these observations indicate that at the mRNA level, EEF1A1 is underexpressed in virtually all breast cancers, but the degree of underexpression is subtype-dependent.

We next evaluated whether EEF1A1 mRNA expression might correlate with specific clinical parameters, including tumor invasion, nodal status, metastasis, stage, age and estrogen receptor (ER), progesterone receptor (PR) and HER2 receptor status. There were no significant associations between EEF1A1 mRNA level and ER, PR or HER2 status (p > 0.05, Mann-Whitney *U* test; Supplementary Fig. S1). However, highly invasive T4 tumors, which invade into other organs, express lower EEF1A1 levels than T1 or T2 tumors, which only invaded into submucosa or muscle, respectively (*p* < 0.05, Mann-Whitney *U* test; Fig. 1E). Additionally, N1/N2/N3 tumors, for which cancer cells have been detected in at least one axillary and/or other nearby lymph node, showed significantly lower EEF1A1 mRNA expression compared to N0 tumors from lymph node-negative patients (*p* < 0.05, Mann-Whitney *U* test; Fig. 1E). Similarly, metastatic breast cancers showed lower EEF1A1 expression than non-metastatic tumors. However, this difference was not statistically significant (p = 0.209), possibly due to low statistical power (n = 19 metastatic tumors; Fig. 1E). We also observed decreased EEF1A1 levels in stage III tumors compared to stage I and stage II breast tumors (p < 0.01, Mann-Whitney *U* test; Fig. 1F). Finally, EEF1A1 levels are reduced in patients over age 54 or post-menopause (p < 0.05, Mann-Whitney *U* test; Fig. 1G), a phenomenon that was confirmed in an independent patient cohort (Fig. 1H). Thus, these observations indicate that EEF1A1 mRNA expression declines with tumor invasion, dissemination to lymph nodes, advanced stage and post-menopause.

### Low EEF1A1 mRNA expression in breast cancer predicts poor patient survival

**Figure 2:**
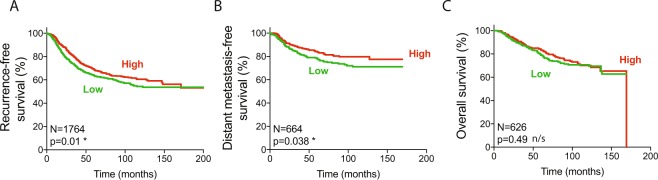
Low *EEF1A1* mRNA expression in breast cancer predicts poor patient survival. (**A**) Recurrence-free survival curve of patients from the Kaplan-Meier plotter dataset. Patients were split into high and low EEF1A1 mRNA expression groups using median expression level as the cut-off, determined as previously described^29^. (**B**) Distant metastasis-free survival curve. (**C**) Overall survival curve. P-values: log-rank Mantel-Cox tests. N/s, not significant; *p < 0.05.

To decipher whether EEF1A1 mRNA expression can predict breast cancer patient prognosis, we first examined the recurrence-free survival (RFS), distant metastasis-free survival (DMFS) and overall survival (OS) from pooled datasets in the Kaplan-Meier plotter online tool using the median expression level as the cut-off between high and low EEF1A1 expressing tumors^29^. This showed that low EEF1A1 mRNA levels are associated with poor RFS and DMSF (p = 0.01 and p = 0.038, log-rank Mantel-Cox test; Fig. 2A,B) but not with OS (Fig. 2C). On the other hand, in the METABRIC dataset, which is about three times larger, we found that low EEF1A1 expression predicted poorer OS (p = 0.0123; Supplementary Fig. S2A).

Using the Kaplan-Meier plotter, we next assessed whether ER status affected the ability of EEF1A1 levels to predict RFS, DMSF and OS. This was not the case for ER+ or for ER- breast cancer patients (Supplementary Fig. S2B). However, somewhat consistent with the association between reduced EEF1A1 levels and increased invasion (Fig. 1E), only for DMFS of ER+ patients, low EEF1A1 levels showed a weak trend towards statistically significant association with poor survival (p = 0.14, n = 161). To explore this further, we studied a much larger combined cohort of 3874 patients, 2822 of whom were ER+ (Table 1)^30^. This indicated that low EEF1A1 mRNA levels correlate with poor DMFS in all breast cancers (p = 3.8 × 10^−5^, log-rank test; Table 1). However, this can entirely be attributed to patients with ER+ tumors (p = 5.7 × 10^−7^), as ER- patients showed no correlation (p = 0.9351; Table 1). Thus, low EEF1A1 levels predict poor breast cancer patient survival, in particular for patients with ER+ tumors.

**Table 1:** Low EEF1A1 mRNA expression is an independent prognostic marker for ER+ breast cancer.

Type	No. of patients	Survival		Univariate			Multivariate^c^			
		DMFS^a^		Prognostic strength			Adjuvant! Online^c^		Nottingham Index^c^	
		P value	P value summary	HR (95% CI)^b^	P value	P value summary	P value	P value summary	P value	P value summary
All	3874	3.8 × 10^−5^	****	0.84 (0.79–0.90)	2.5 × 10^−7^	****	0.0262	*	0.0893	n/s
ER+	2822	5.7 × 10^−7^	****	0.78 (0.72–0.85)	9.0 × 10^−9^	****	0.0009	***	0.0329	*
ER−	1022	0.9351	n/s	0.99 (0.88–1.12)	0.8796	n/s	0.5355	n/s	0.6672	n/s

^a^Distant metastasis-free survival (DMFS) using log-rank tests. ^b^HR, hazard ratio; CI, confidence interval. ^c^Calculated as previously described^31,32^.

### Low EEF1A1 mRNA expression is an independent marker for poor prognosis of ER+ breast cancer

It is well established that differential gene expression in breast cancer is often associated with key clinical parameters, such as tumor size and lymph node status. Therefore, it is critical to assess whether any potential marker retains prognostic strength independent of such variables using multivariate analyses. To test this, we first fitted a univariate Cox proportional hazard model on the combined 3874 patients from 26 independent datasets^30^ (Supplementary Table S1). This again indicated that EEF1A1 underexpression is a strong marker for poor prognosis for all breast cancers combined (Hazard Ratio (HR) = 0.84, 95% Confidence Interval (CI) = 0.79–0.90, p = 2.5 × 10^−7^; Table 1). However, this is solely due to patients with ER+ breast cancers (HR = 0.78, 95%CI = 0.72–0.85, p = 9.0 × 10^−9^; Table 1), as there is no univariate prognostic strength for ER- breast cancer patients (HR = 0.99, 95%CI = 0.88–1.12, p = 0.8796; Table 1).

To test whether EEF1A1 expression can independently predict patient prognosis, we applied multivariate analyses using clinical parameters included in Adjuvant! Online and the Nottingham Prognostic Index^31,32^. Adjuvant! Online is a computer program, which accounts for clinical parameters to estimate the risk of poor patient outcome in adjuvant therapy. The Nottingham Prognostic Index takes tumor size, grade and nodal status into account for post-surgery outcome prediction. Using the same combined datasets, following adjustment to these well-established concepts, we stringently determined in multivariate analyses that EEF1A1 mRNA underexpression is an independent prognostic biomarker for ER+ (p = 9.0 × 10^−9^ and p = 0.0329) but not ER- breast cancers (p = 0.8796, p = 0.6672; Table 1).

### Low EEF1A1 mRNA expression in breast carcinoma is not due to *EEF1A1* mutations or hypermethylation of its promoter

**Figure 3:**
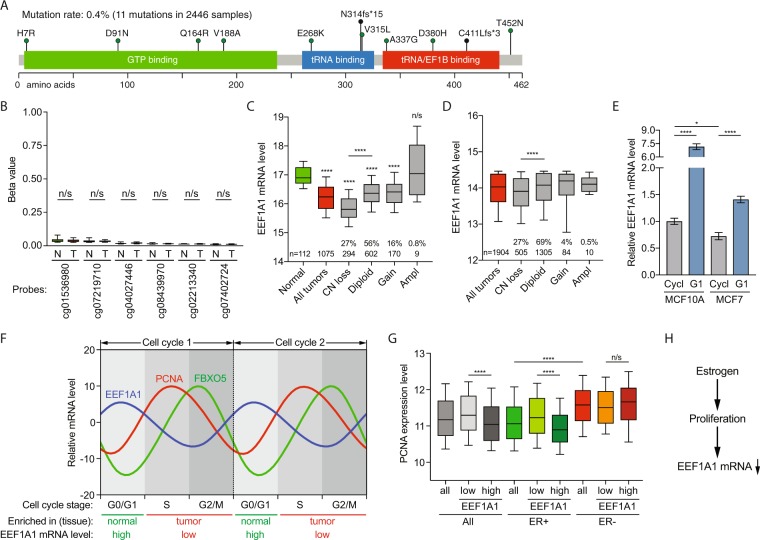
Low EEF1A1 mRNA expression in breast carcinoma is due to *EEF1A1* allelic copy number loss and cell cycle-associated expression. (**A**) Mutations identified in 2,446 breast cancer samples from the COSMIC database (version 83, see Methods). The image was generated as described^56,57^ and modified. Scale bar indicates amino acid numbers. (**B**) Box plot of β-values of the six CpG probes in the *EEF1A1* promoter 1000 base pairs upstream of the EEF1A1 transcription start site in normal breast tissue (N) and breast tumor (T). Data are derived from TCGA. P-values: Mann-Whitney *U* test; n/s, not significant. (**C**) Box plot of EEF1A1 mRNA expression level in breast carcinomas and normal breast tissue for indicated *EEF1A1* allelic copy number status. Data are derived from the TCGA RNAseq and SNP6 microarray datasets^26^. P-values: Mann-Whitney *U* test. (**D**) Box plot as in (**B**) but using data from the METABRIC datasets^27,28^. P-values: Mann-Whitney *U* test. N/s, not significant; ****p < 0.0001. (**E**) Bar graph of EEF1A1 mRNA expression levels in asynchronously growing/cycling and serum-starved/G1-arrested MCF10A and MCF7 cells, as determined by qRT-PCR. Data are normalized to cycling MCF10A cells. P-values: Student t-test. *p < 0.05; ****p < 0.0001. (**F**) Graph showing oscillating, cell cycle stage-dependent mRNA expression levels of EEF1A1, the S-phase marker PCNA and the G2/M marker FBXO5^36^. (**G**) Box plot of PCNA expression levels in breast carcinomas with indicated estrogen receptor (ER) status. Tumors were split into high and low EEF1A1 mRNA expression groups using median expression level as the cut-off. Data are derived from TCGA. P-values: Mann-Whitney *U* test. N/s, not significant; ****p < 0.0001. (**H**) Model for the relationship between estrogen receptor signaling and EEF1A1 mRNA expression in ER+ breast cancers.

Next, we assessed whether mutations in *EEF1A1* could account for the reduced EEF1A1 mRNA expression in breast cancers. However, in a combination of multiple breast cancer datasets (see Methods and Supplementary Table S1), only 11 mutations were identified in 2,446 tumor samples (Fig. 3A). This low mutation rate of 0.4% could not explain the broad underexpression of EEF1A1 observed in breast cancer.

To test whether *EEF1A1* promoter methylation could explain the low mRNA expression, we compared the *EEF1A1* promoter methylation level between normal breast tissue and breast carcinoma using the TCGA dataset^26^. However, we find that the CpG methylation status measured for all CpG probes that were located in the 1000 base pairs upstream of the *EEF1A1* transcription start site were very low in both normal breast tissue and breast carcinoma tissue and these minimal differences did not significantly change (Fig. 3B), indicating that low EEF1A1 mRNA expression is not caused by hypermethylation of the *EEF1A1* promoter.

### Low EEF1A1 mRNA expression is due to EEF1A1 allelic copy number loss and cell cycle-associated expression

To test if *EEF1A1* allelic copy number loss could contribute to low mRNA expression, we used the TCGA RNAseq dataset for mRNA expression and the SNP6 microarray dataset for somatic copy number aberrations^26,33^. *EEF1A1* allelic copy number loss occurs in 27% of breast tumors, and this is significantly associated with reduced EEF1A1 mRNA expression compared to *EEF1A1* diploid tumors (p < 0.0001, Mann-Whitney *U* test; Fig. 3C). Similarly, in the METABRIC dataset, 27% of breast cancers showed *EEF1A1* copy number loss and these tumors also expressed significantly lower EEF1A1 than tumors diploid for *EEF1A1* (p < 0.0001; Fig. 3D). However, EEF1A1 mRNA expression is also significantly lower in diploid tumors than in normal tissue (p < 0.0001; Mann-Whitney *U* test; Fig. 3C). Thus, *EEF1A1* allelic copy number loss only partly accounts for the observed EEF1A1 mRNA underexpression.

Compared to normal cells, tumor cells are highly proliferative. Hence, relative to normal tissues, tumor tissues contain higher fractions of cells in S/G2/M stages of the cell cycle^34,35^. As this could potentially explain why EEF1A1 mRNA levels are reduced in breast carcinoma, we thus asked whether EEF1A1 mRNA expression is cell cycle-associated. To this end, we synchronized the breast cancer cell lines MCF10A and MCF7 in G1 stage of the cell cycle using serum-free media. Real-time quantitative reverse-transcription PCR (qRT-PCR) showed that EEF1A1 mRNA levels are significantly upregulated in these G1-synchronized cells compared to respective asynchronously growing MCF10A and MCF7 cells (each p < 0.0001, unpaired t-test; Fig. 3E). In addition, the EEF1A1 mRNA level in more proliferative MCF7 cells is lower than in non-cancerous and less proliferative MCF10A cells (p < 0.05, unpaired t-test; Fig. 3E). This indicates that EEF1A1 mRNA levels are higher in cells in G1 stage than in cycling/proliferating cells.

Consistently, an independent approach^36^ shows that the relative EEF1A1 mRNA levels are 5.4, −1.2 and −5.7 during G1, S and G2/M stages of the cell cycle, respectively. This further confirms that EEF1A1 mRNAs oscillate during cell cycle progression, with these being the highest at G1 stage (Fig. 3F). Since, compared to normal tissues, tumor tissues are enriched for cells in S/G2/M stage of the cell cycle (Fig. 3F), these data strongly suggest that the G1-specific peak levels of EEF1A1 mRNA predominantly account for our observation that EEF1A1 mRNA levels are reduced in breast carcinoma.

Our above observations prompted us to also further explore a potential link between EEF1A1 mRNA levels and ER signaling. It is well-established that estrogen stimulates breast cancer cell proliferation^37,38^. Consistently, in ER+ tumors ER activity is higher in EEF1A1-low than in EEF1A1-high tumors, but in ER- tumors there is no significant difference (Supplementary Fig. S4). Further in line with this, while ER- tumors are more proliferative than ER+ tumors, as measured by the expression levels of the S phase-specific proliferation marker PCNA (Fig. 3F,G), EEF1A1 mRNA-low tumors are more proliferative than EEF1A1 mRNA-high tumors in ER+ but not in ER- breast cancers (Fig. 3G). Strikingly, these data closely resemble our earlier observation that low EEF1A1 mRNA levels are an independent marker for poor patient survival for ER+ but not for ER− breast cancer patients (Table 1). These results further suggest that EEF1A1 mRNA levels are reduced in ER+ breast tumors due to ER-induced cell proliferation (Fig. 3H).

### At the protein level, eEF1α1 is overexpressed in ductal breast carcinoma

We next asked whether EEF1A1 is also underexpressed in breast carcinomas at the protein level. To that end, we performed immunohistochemistry (IHC) on a tissue microarray, which included 7 adjacent normal breast tissues and 100 ductal breast carcinomas, using a previously well-characterized antibody^39–41^. Clinicopathological features and prognostic variables of the patients whose samples were analyzed are included in Supplementary Table S2.

**Figure 4:**
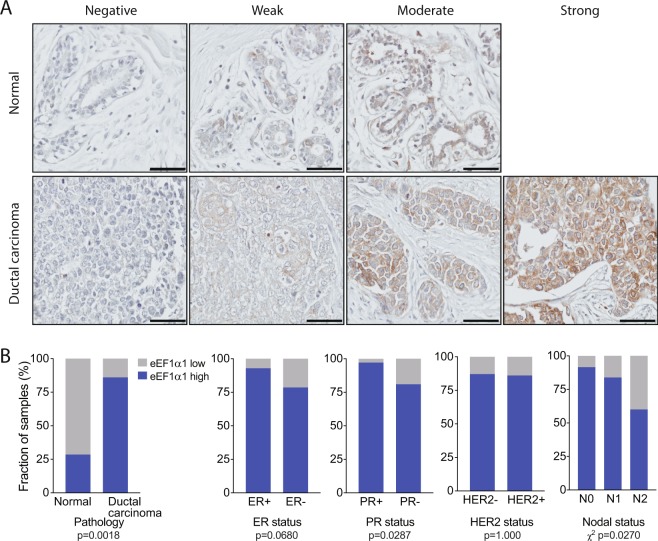
Immunohistochemistry shows that eEF1α1 protein is overexpressed in ductal breast carcinoma. (**A**) A tissue microarray with a total of 7 adjacent normal breast tissues and 100 ductal breast carcinomas was immunohistochemically stained with an anti-eEF1α1 antibody. Representative images of various staining intensities are shown. Scale bars: 50 μm. (**B**) Distributions of high and low eEF1α1-expressing tissue samples, using an immunohistochemistry-derived H-score of 50 as a cutoff. Data are analyzed for several clinical parameters, as indicated. P-values: Fisher’s exact tests or Chi-square tests, as indicated.

Following an optimized IHC protocol (see Methods), we found that the staining intensities of the samples ranged from negative to moderate in normal breast tissues and from negative to strong in breast carcinomas (Fig. 4A). Interestingly, in sharp contrast to our observations at the mRNA level, at the protein level eEF1α1 seemed to be overexpressed in these ductal breast carcinomas compared to normal breast tissues. To stringently test this, we assigned H-scores to each sample using a multiplicative IHC quick-score method (see Methods). H-scores account for both the staining intensity and the fraction of positive cells, ranging from 0 (negative) to 300 for all epithelial cells showing strong staining. First, using an H-score of 50 as a cut-off, we found that high eEF1α1 expression was significantly more common in ductal carcinoma (86 of 100, 86%) than in normal breast tissue (2 of 7, 29%; p = 0.0018, Fisher’s exact test; Fig. 4B, Table 2).

**Table 2:** EEF1α 1 expression in normal breast and ductal breast carcinoma tissues.

Variable	Number of samples with H-score >50/total (%)	*p* value vs normal^a,b^	Other (*p* value)^a,b^
All samples			
Normal breast	2/7 (29)		
Ductal carcinoma	86/100 (86)	**0**.**0018**	
Grade			
2	46/54 (85)	**0**.**0035**	
3	36/42 (86)	**0**.**004**	
Tumor invasion			
T1	8/10 (80)	0.0584	
T2	55/62 (89)	**0**.**0012**	
T3	14/16 (88)	**0**.**0107**	
T4	9/12 (75)	0.0739	
Nodal status			
N0	54/59 (92)	**0**.**0005**	N0 vs N2 (**0**.**0207**)
N1	26/31 (84)	**0**.**008**	N0/N1 vs N2 (**0**.**0316**)
N2	6/10 (60)	0.3348	
Metastasis			
M0	86/99 (88)	**0**.**0014**	
M1	0/1 (0)	1.000	
Estrogen Receptor status			
ER+	52/56 (93)	**0**.**0003**	ER+ vs ER− (0.068)
ER−	33/42 (79)	**0**.**0151**	
Progesterone Receptor status			
PR+	34/35 (97)	**0**.**0001**	PR+ vs PR− (**0**.**0287**)
PR−	51/63 (81)	0.077	
HER2 receptor status			
HER2+	31/36 (86)	**0**.**0043**	HER2+ vs HER2− (1.0)
HER2−	54/62 (87)	**0**.**0019**	
Age/Menopausal status			
<48	46/56 (82)	**0**.**0066**	
48–54	23/25 (92)	**0**.**0019**	
>54	17/19 (89)	**0**.**0057**	

^a^Calculated using Fisher’s exact test.

^b^*P* values in bold remain statistically significant at a false discovery rate (FDR) of 0.05.

Second, we assessed whether EEF1A1 protein expression specifically associated with several clinical parameters. High eEF1α1 expression occurred in more ER+ than ER- tumors (93% versus 79%) and this difference approached statistical significance (p = 0.0680; Fig. 4B). Significantly more PR+ tumors expressed high eEF1α1 (97% versus 81%, p = 0.0287; Fig. 4B). However, we did not observe any significance between HER2+ and HER2− tumors (86% versus 87%, p = 1; Fig. 4B). In addition, eEF1α1 expression in N0 tumors is significantly higher than in N1/N2 tumors (p = 0.0207, Chi-square test; Fig. 4B), but there are no significant differences based on age (p = 0.4427), metastatic state (p = 0.1400), tumor invasion (p = 0.5907) or tumor grade (p = 1.0; Supplementary Fig. S3A).

Finally, despite the fact that there is no significant association between thresholded H-scores and age groups, as a continuous variable the H-score of eEF1α1 expression is significantly increased in patients over age 54 (p < 0.05, Mann-Whitney *U* test; Supplementary Fig. S3B). Taken together, at the protein level, eEF1α1 is significantly overexpressed in PR+ and lymph node-negative ductal breast carcinomas, as well as in tumors of patients over 54 years of age.

### EEF1α1 protein expression levels parallel cellular stress levels

**Figure 5:**
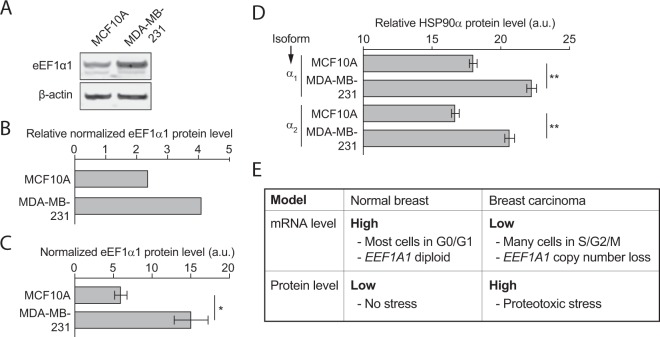
EEF1α1 protein expression levels parallel cellular stress levels. (**A**) Western blots showing eEF1α1 and β-actin protein levels in MCF10A and MDA-MB-231 breast cancer cell lines. (**B**) Quantification of β-actin-normalized eEF1α1 protein levels in MCF10A and MDA-MB-231 cell lines using the Western blots shown in (A). (**C**) Bar graph of normalized eEF1α1 protein levels in MCF10A and MDA-MB-231 cell lines using mass spectrometric quantification (each n = 2)^42^. (**D**) Protein expression levels of the heat shock response-inducible HSP90 isoforms α_1_ and α_2_. (**E**) Model explaining the contradictory EEF1A1 mRNA and eEF1α1 protein misexpression in breast carcinoma.

To investigate the role of elevated eEF1α1 protein expression in breast cancer, we first performed Western blot analysis on breast cancer cell lines. We found that eEF1α1 expression is higher in MDA-MB-231 breast cancer cells than in non-cancerous MCF10A cells (Fig. 5A,B, Supplementary Fig. S5). Consistently, mass spectrometric determination of protein levels in these cell lines^42^ also independently showed significantly higher eEF1α1 protein levels in MDA-MB-231 cells compared to MCF10A cells (Fig. 5C).

A recent study showed that eEF1α1 strongly promotes the heat shock response^43^, which is induced following proteotoxic stress, caused by for instance thermal shock, oxidative stress or aneuploidy. In line with this, compared to non-tumorigenic MCF10A cells, increased eEF1α1 protein levels in cancerous MDA-MB-231 cells are paralleled by increased protein expression of both the α_1_ and α_2_ isoforms of the stress-induced heat shock protein HSP90 (Fig. 5D).

## Discussion

Chromosome instability (CIN) may occur via a range of mechanisms, including cell cycle checkpoint defects, aberrant DNA repair, failed DNA replication and mitotic dysregulation, and CIN is common in solid cancers due to p53 or RB pathway defects^5,7,8,44,45^. Copy number alterations in particular result in dramatically imbalanced proteome homeostasis and heterogeneity within the tumor and this is associated with poor patient outcome and multi-drug resistance^13–15^. CIN impairs protein folding, induces proteotoxic stress and may trigger the heat shock response (HSR)^13–15,46,47^. Our interest in CIN and its consequences led us to study eEF1α1, which was recently shown to strongly promote the HSR^43^.

We find that EEF1A1 mRNA is underexpressed in advanced breast cancers. Consistently, our qRT-PCR analyses show lower EEF1A1 mRNA levels in the breast cancer cell line MCF7 than in non-cancerous MCF10A cells. Underexpression occurs in particular in invasive, lymph node-positive, advanced stage and postmenopausal tumors, suggesting that EEF1A1 mRNA levels typically decline as breast cancers become more malignant. Using stringent multivariate analyses, which account for various other clinical variables, we also demonstrate that low EEF1A1 mRNA is an independent prognostic marker for ER+ but not for ER− breast cancer patients.

We studied four potential mechanisms that could explain reduced EEF1A1 mRNA expression in breast cancer. We essentially ruled out *EEF1A1* mutations and promoter hypermethylation, as they are very rare or non-existent. However, *EEF1A1* allelic copy number loss occurs in 27% of tumors and this significantly reduced EEF1A1 mRNA levels compared to diploid tumors. Yet, as diploid tumors also showed significantly lower EEF1A1 expression levels than normal breast tissues, this can only partly explain the widespread EEF1A1 mRNA underexpression. Indeed, we believe that cell cycle-associated oscillation of EEF1A1 mRNA levels, which peak in G1 phase, is a major cause. Our cell synchronization experiments and microarray data both show significantly decreased EEF1A1 mRNA levels in proliferating cells. Tumors are proliferative and therefore enriched in cells in S/G2/M phases of the cell cycle compared to normal tissues with most cells in G0/G1 phase^34,35^. Thus, G1 phase-specific EEF1A1 mRNA expression seems the major cause of underexpression in breast cancers (Fig. 5E).

Interestingly, this finding also provides an explanation for why EEF1A1 mRNA underexpression is prognostic for ER+ tumors only. Consistent with the above, ER+ tumors with low EEF1A1 mRNA expression show higher expression of the proliferation marker PCNA compared to ER+ tumors with high EEF1A1 mRNA expression. However, this difference is not observed for ER- breast cancers. Since estrogen has been shown to promote breast cancer proliferation^37,38^, this suggests a direct relationship between estrogen signaling and EEF1A1 mRNA expression (Fig. 3H).

In sharp contrast to reduced EEF1A1 mRNA expression, we observed that at the protein level, eEF1α1 is overexpressed in breast carcinomas, in particular in ER+, PR+ and lymph node-negative tumors. This is consistent with observations in hepatocellular carcinomas^48,49^. We also find that in breast cancer cell lines, eEF1α1 levels correlate with protein levels of HSP90α_1_ and HSP90α_2_, which are markers of the heat shock response (HSR). This is in line with other recent findings, including that eEF1α1 strongly promotes the HSR^43,50^. Importantly in fact, reduction of high eEF1α1 protein levels by short hairpin RNAs was shown to, under stress conditions, protect MDA-MB-231 cells from cell death^43^, thus providing a malignant advantage to breast cancer cells. Collectively, this indicates that eEF1α1 protects breast cancer cells from stress-induced cell death by promoting the HSR. It may do so by recruiting HSF1, the master regulation of the HSR, to critical promoters^43^, which may subsequently stabilize oncoproteins, such as mutant p53^18^.

The discrepancy between the direction of EEF1A1 mRNA and protein misexpression (Fig. 5E) suggests strong post-transcriptional regulation. For this, mRNA regulatory elements and the affinity of RNA Binding Proteins (RBPs) could be important. Both post-transcriptional mechanisms have been linked to enhanced mRNA stability and translational efficiency of mRNA molecules, leading to abnormal protein overexpression in tumor cells^51^. Alternatively, post-translational modifications of eEF1α1 could markedly stabilize eEF1α1 proteins and prevent their degradation.

Taken together, we observe contradictory mRNA and protein misexpression of EEF1A1 in ductal breast carcinoma. However, both are significantly associated poor patient outcome. Cell cycle-associated EEF1A1 mRNA expression and the HSR, protecting tumor cells from stress-induced cell death, explain the opposite direction of misexpression and indicates strong post-translational control of eEF1α1 protein levels in tumors.

## Methods

### Gene expression analyses

EEF1A1 mRNA expression levels were evaluated in a range of previously published datasets (Supplementary Table S1)^25^. For in-depth analyses, EEF1A1 mRNA expression values from The Cancer Genome Atlas (TCGA) Illumina HiSeq2000 RNA sequencing breast cancer dataset were used (Supplementary Table S1)^26,33^. Level 3 log2-normalized EEF1A1 mRNA expression levels were used for comparisons between normal and tumor samples and/or between samples of different breast cancer subtypes, age, menopause status, stages, hormone receptor status (ER, PR, and HER2), tumor invasion, lymph node status, and metastatic state. Similar comparisons were performed using the METABRIC dataset^27,28^. In the analyses, normal tissues refer to healthy tissues from cancer patients. Matched statistical analyses were performed as described^52^. As indicated, Mann-Whitney *U* tests or Wilcoxon matched-pairs signed rank tests were used to assess whether the differences were statistically significant.

### Somatic mutation analysis

Breast cancer-specific EEF1A1 somatic mutations were identified using 2,446 samples from the COSMIC database (version 83; Supplementary Table S1)^53^. Most of these samples were from the TCGA whole exome sequencing (n = 982), International Cancer Genome Consortium (ICGC) (n = 569) and INSERM (n = 213) breast cancer datasets^33,54,55^. Mutation data were visualized as described^56,57^ but with minor modifications, as indicated.

### DNA methylation analysis

*EEF1A1* promoter methylation levels were determined from Illumina Infinium HumanMethylation450 platform level 3 TCGA data^26^, as described^58^, using the β-values for all CpG probes in the region between positions −1000 and +1 base pairs with respect to the *EEF1A1* transcription start site.

### Somatic copy number alteration analyses

*EEF1A1* allelic somatic copy number alterations (SCNAs) were analyzed and processed as previously described^58^. Briefly, Affymetrix Genome-Wide SNP6.0 Array data were obtained from the TCGA breast cancer studies^26,33^. These were processed using GISTIC2.0 and allelic copy number status (loss, diploid, gain or amplification) was determined for each sample.

### Survival analyses

Survival analyses were performed either using the Kaplan-Meier Plotter tool^29^ or using Illumina HT-12v3 microarray gene expression data from the METABRIC study^27,28^. Patients were grouped into low and high expression, using the median expression as the cut-off, as described^30^. Survival curves were re-plotted in GraphPad Prism. For all comparisons of survival curves, log-rank Mantel-Cox tests were used to assess statistical significance.

### Clinical prognostic analyses

The strength of EEF1A1 expression as a prognostic marker, in all breast cancers and in ER+ and ER− tumors separately, was determined using univariate an multivariate Cox proportional hazard analyses, as described^11^. Briefly, reported univariate hazard ratios (HRs) and 95% confidence intervals (CIs) only assessed EEF1A1 expression. For multivariate analyses, clinical parameters included in Adjuvant Online!^31^ and the Nottingham Prognostic Index^32^ were included as co-variates to determine the respective HRs with 95% CIs.

### Tissue samples, ethics statement and immunohistochemistry

Tissue samples on the tissue microarray (TMA) slides were obtained under Health Insurance Portability and Accountability Act (HIPAA)-approved protocols, in accordance with the approved guidelines and with informed consent from the donors. With ethical approval from the Medical Research Ethics Committee (MREC) at the University of Queensland, immunohistochemistry was performed as described^12^, with modifications. Breast cancer TMA slides (US Biomax) were baked at 60 °C for 30 minutes, de-paraffinized with xylene at room temperature (RT) for 3 min, 2 min and 2 min each and rehydrated by ethanol series 100%, 90%, 70%, each twice for 2 min and once for 2 min in ddH_2_O. Antigen retrieval was followed by incubating slides with sodium citrate buffer (10 mM sodium citrate, 0.05% Tween-20, pH6) at 85 °C for 20 min in a pressure cooker. Slides were cooled at RT for 20 min, washed with TBS three times and incubated with 0.3% H_2_O_2_ in TBS for 10 min to block endogenous peroxidase activity. After three TBS washes, slides were incubated with blocking buffer (MACH 1™, Biocare Medical) for 10 min, with rabbit anti-human eEF1α1 primary antibody (Proteintech, #11402-1-AP) in 1:800 dilution for 2 hours at RT, washed with TBS three times and incubated with secondary antibody (HRP-polymer, MACH 1™, Biocare Medical) for 30 min at RT. Next, slides were washed with TBS three times, 3,3′-diaminobenzidine (DAB) was applied as the chromogen and hematoxylin was used to counterstain nuclei. Tissues on slides were dehydrated in backwards ethanol series (70%, 90%, 100%) for 2 min each and slides were heated to 60 °C to dry. Finally, slides were incubated with xylene for 2 min and coverslips and Permount mounting medium (Fisher Scientific) were applied.

Images were acquired using Virtual Slide Microscope (VS120, Olympus). Slides were independently scored by two individuals and in a blinded fashion. A multiplicative IHC quick-score method was applied as follows. The average intensity of the staining (on a scale of 0 to 3) was multiplied by the proportion of positively stained epithelial/tumor cells in the section (on a scale of 0 to 100%)^59^. Clinical endpoints examined are included in Table 2. H-scores of ≤50 and >50 were considered low and high in eEF1α1 expression, respectively. Fisher’s exact and Chi-square tests were used to assess whether differences were statistically significant.

### Cell culture and synchronization

MCF10A cells were cultured in Dulbecco’s Modified Eagle Medium: Nutrient Mixture F-12 (DMEM/F12) with 5% horse serum, 20 ng/ml rhEGF, 500 ng/ml hydrocortisone, 100 ng/ml cholera toxin, 0.01 mg/ml insulin and 1% penicillin/streptomycin (p/s). MCF7 cells were cultured in DMEM with 10% fetal bovine serum, 0.01 mg/ml insulin, 1% L-glutamine +1% sodium pyruvate and 1% p/s. MDA-MB-231 cells were cultured in the same medium but without insulin. For G1 phase cell cycle synchronization, cells were cultured in serum-free media containing 1 mg/ml BSA for 24 hours.

### Western blot analysis

Cells were grown in 25T-flasks and collected at 70–80% confluence. Cells were lysed in RIPA buffer (150 mM sodium chloride, 1% Triton X-100, 0.5% sodium deoxycholate, 0.1% SDS, 50 mM Tris-HCl at pH 8.0 and 1:400 dilution of Protease Inhibitors Cocktail (P8340, Sigma)). Proteins were separated in 8–20% Tris-Acetate gels at 100 V for 2 hr and wet-transferred onto the Immunobilin-FL PVDF membranes (0.45μm pore size, Merck Millipore) at 90 V and 4 °C for 2 hr using Mini Trans-Blot® Electrophoretic Transfer Cell (Bio-rad). Membranes were blocked with TBS-based Odyssey blocking buffer (Li-COR) for 1 hr, followed by overnight incubation with primary antibodies rabbit anti-human eEF1α1 (Proteintech, #11402-1-AP; 1:1000 dilution) and mouse anti-human β-actin (SC-47778, Santa Cruz) at 4 °C. Membranes were washed four times with TBS-T (0.05% of Tween-20) for 5 min each, incubated with secondary antibodies (Li-COR, 680-red or 800-green) for 1 hr at RT and scanned and quantified on an Odyssey CLX imager (Li-COR). Normalized eEF1α1 protein levels were determined by dividing eEF1α1 levels by β-actin levels.

### Real-time quantitative reverse-transcription PCR (qRT-PCR)

Total RNA was isolated with Trizol reagent (Invitrogen) according to manufacturer’s guidelines. Reverse transcription was performed with Tetro cDNA Synthesis Kit (Bioline) according to manufacturer’s description. Quantitative PCR was performed with SYBR-green PCR master mix using QuantStudio™ 7 Flex Real-Time PCR Instrument (both Thermo Fisher Scientific) with cycling conditions recommended by the manufacturer. The EEF1A1 raw PCR cycles (CT) values were normalized (ΔCT) to internal control (hypoxanthine phosphoribosyltransferase 1; HPRT1) CT values. Relative mRNA level changes were determined by 2^−ΔΔCT^ calculation using MCF10A asynchronous cells as controls. Primers used were: HPRT1-forward: TCAGGCAGTATAATCCAAAGATGGT, reverse: AGTCTGGCTTATATCCAACACTTCG^60^; EEF1A1-forward: GGACACGTATCGGGCAA, reverse: AGGAGCCCTTTCCCATCTCA.

## Electronic supplementary material

Supplementary Information

## Data Availability

All datasets analyzed in the current study are available via the links and references included in Supplementary Table S1.
