# Supplementary material for: Contradictory mRNA and protein misexpression of EEF1A1 in ductal breast carcinoma due to cell cycle regulation and cellular stress
Source: Sci Rep. 2018 Sep 17;8:13904. doi: 10.1038/s41598-018-32272-x (PMC6141510; doi:10.1038/s41598-018-32272-x)
Supplement: Supplementary file 1 — Supplementary Information [file 41598_2018_32272_MOESM1_ESM.pdf]

Supplementary Information

Contradictory mRNA and protein misexpression of EEF1A1 in ductal breast carcinoma due to cell cycle regulation and cellular stress

Cheng-Yu Lin, Alexandra Beattie, Behzad Baradaran, Eloise Dray, Pascal HG Duijf

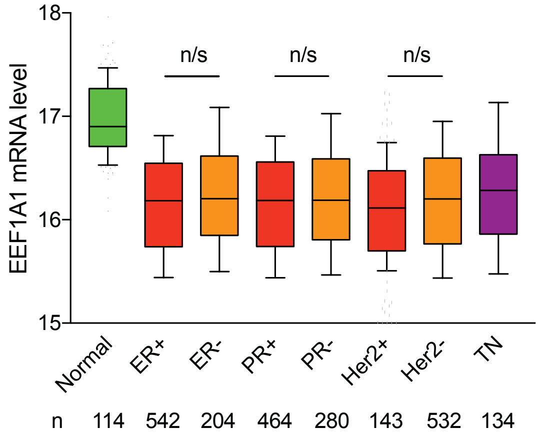

Figure S1.
Box plot showing normalized EEF1A1 mRNA expression in breast tumors for indicated receptor statuses. Analyzed data are from The Cancer Genome Atlas (TCGA) breast carcinoma RNAseq dataset. TN, triple negative breast cancer; n/s, not significant.

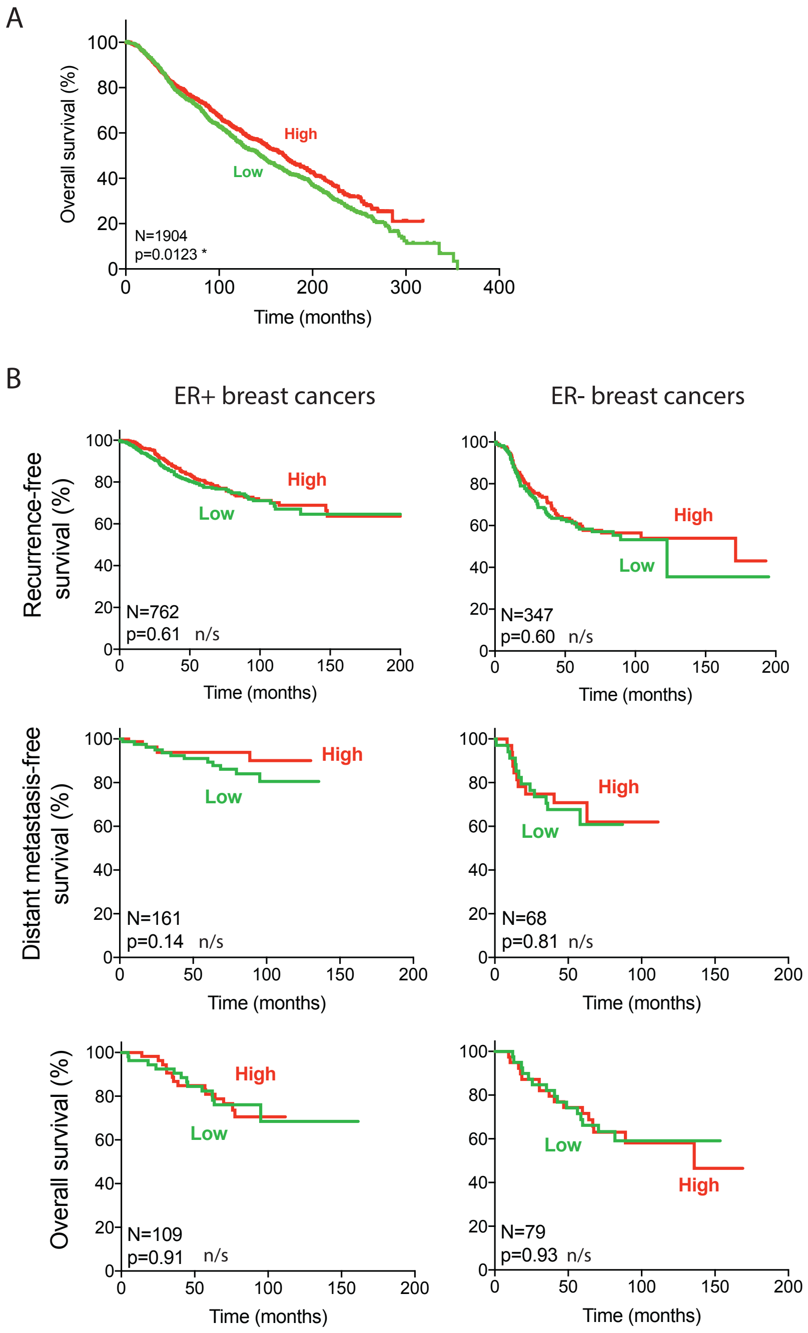

Figure S2.
(A) Overall survival curves of patients from the METABRIC dataset. Patients whose tumors showed high or low EEF1A1 mRNA expression were split using the median expression level as the cutoff. P-value: log-rank Mantel-Cox test.
(B) Recurrence-free survival, distant metastasis-free survival and overall survival curves of patient from the combined Kaplan-Meier plotter datasets. Patients were examined according to their breast tumor's estrogen receptor status, ER+ or ER-. P-values: log-rank Mantel-Cox tests. N/s, not significant.

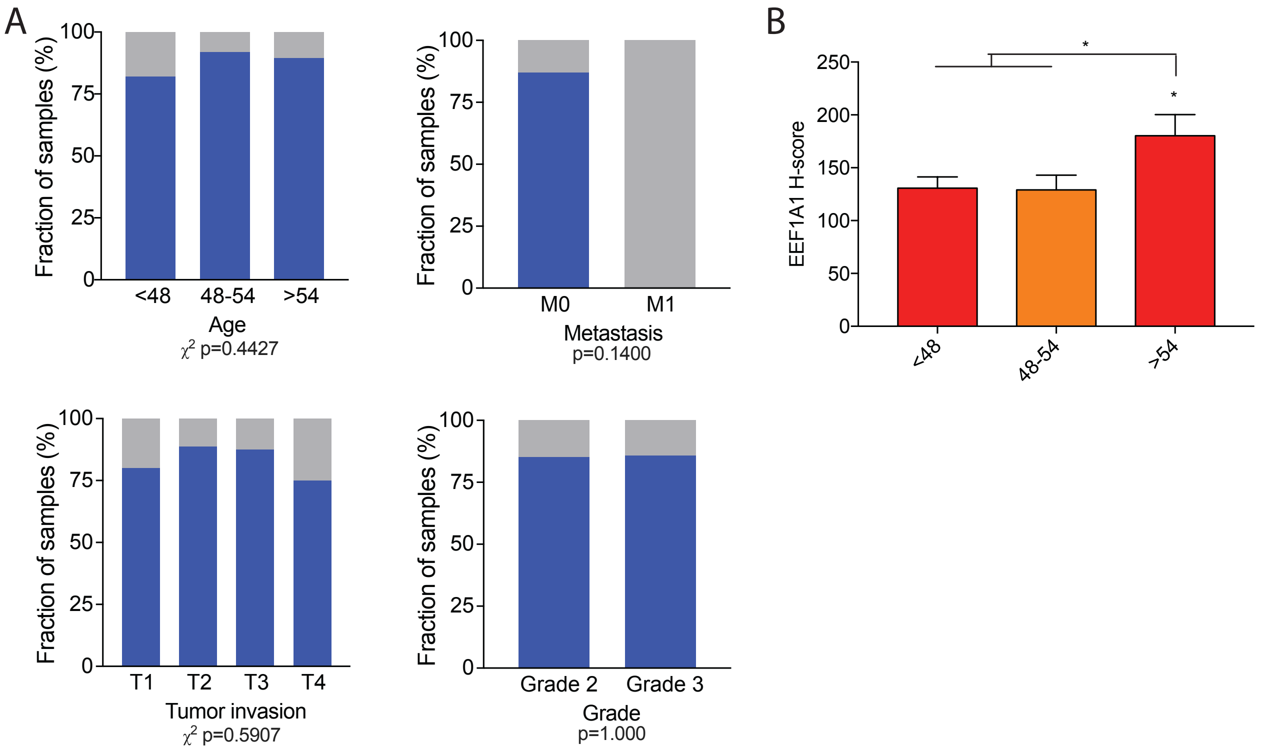

Figure S3.

(A) Distribution of the numbers of eEF1α1 high and low expression samples, based on immunohistochemistry-derived H-scores, by indicated clinical parameter. P-values: Fisher's exact tests or Chi-square tests, as indicated.

(B) Bar graph of eEF1α1 H-score in breast tumors grouped by age. P-value: Mann-Whitney *U* test. *, p<0.05.

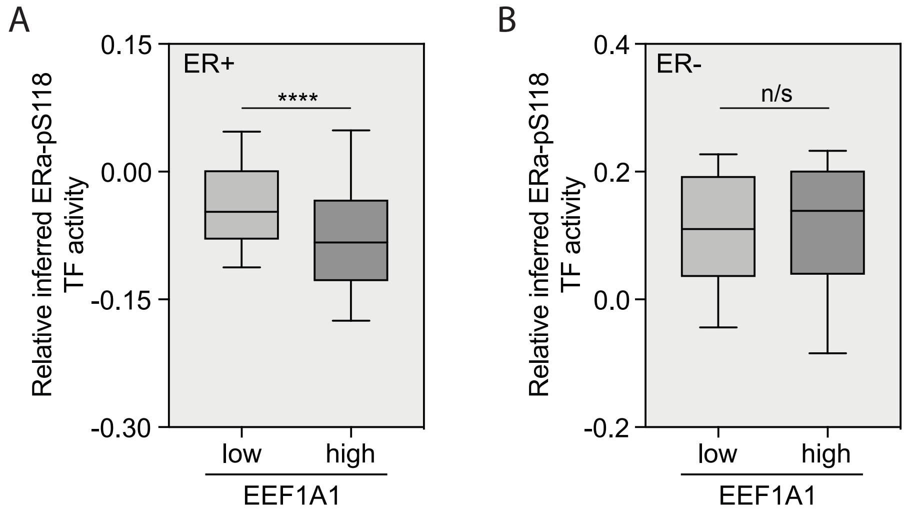

Figure S4.

Inferred estrogen receptor (ER) transcription factor (TF) activity, as determined for ER-phospho-Ser118 (ERa-pS118) using a previously described affinity regression algorithm (Osmanbeyoglu et al., 2014), for ER+ (A) and ER- (B) breast carcinomas, respectively. Samples were split into EEF1A1 mRNA-low and -high using the median EEF1A1 mRNA level as a cutoff. P-values: Mann-Whitney *U* test. ****, p<0.0001; n/s, not significant.

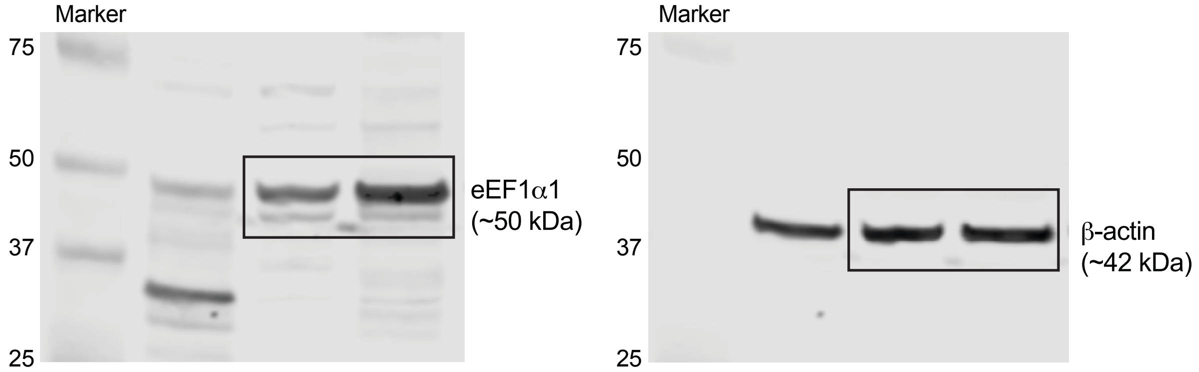

Figure S5.
Uncropped Western blots shown in main Figure 5A. Molecular weights of the bands in the marker lanes are shown on the left in kilodaltons.

Table S1. Details and availability of datasets used in this study

Study no.	Dataset reference	PubMed ID	Dataset ID/ availability [a]	Used for analysis in	Number of samples
1	Cancer Genome Atlas, 2012	23000897	https://portal.gdc.cancer.gov	Fig. 1a,b,d-g, 3a-c,g	593/1,011
2	Curtis et al., 2012	22522925	EGAS00000000083	Fig. 1a,c,h, 3d	2,136
3	Radvanyi et al., 2005	16043716	GSE1477	Fig. 1a	63
4	Richardson et al., 2006	16473279	GSE3744	Fig. 1a	47
5	Ma et al., 2009	19187537	GSE14548	Fig. 1a	66
6	Karnoub et al., 2007	17914389	GSE8977	Fig. 1a	22
7	Zhao et al., 2004	15034139	GSE3971	Fig. 1a	64
8	Gluck et al., 2012	21373875	GSE22358	Fig. 1a	158
9	Finak et al., 2008	18438415	GSE9014	Fig. 1a	59
10	Perou et al., 2000	10963602	http://genome-www.stanford.edu/breast_cancer/molecularportraits	Fig. 1a	65
11	Turashvili et al., 2007	17389037	GSE5764	Fig. 1a	30
12	Guedj et al., 2012	21785460	E-MTAB-365	Fig. 2a-c	537
13	Bertheau et al., 2007	17388661	E-TABM-43	Fig. 2a-c	37
14	Schmidt et al., 2008	18593943	GSE11121	Fig. 2a-c	200
15	Zhang et al., 2009	18821012	GSE12093	Fig. 2a-c	136
16	Bos et al., 2009	19421193	GSE12276	Fig. 2a-c	204
17	Pawitan et al., 2005	16280042	GSE1456	Fig. 2a-c	159
18	Desmedt et al., 2009	19573224	GSE16391	Fig. 2a-c	55
19	Desmedt et al., 2011	21422418	GSE16446	Fig. 2a-c	120
20	Shi et al., 2010	20676074	GSE16716	Fig. 2a-c	47
21	Symmans et al., 2010	20697068	GSE17705	Fig. 2a-c	196
22	Sircoulomb et al., 2010	20932292	GSE17907	Fig. 2a-c	54
23	Korde et al., 2010	20012355	GSE18728	Fig. 2a-c	61
24	Li et al., 2010	20098429	GSE19615	Fig. 2a-c	115
25	Popovici et al., 2010	20064235	GSE20194	Fig. 2a-c	45
26	Tabchy et al., 2010	20829329	GSE20271	Fig. 2a-c	96
27	Wang et al., 2005	15721472	GSE2034	Fig. 2a-c	286
28	Kao et al., 2011	21501481	GSE20685	Fig. 2a-c	327
29	Dedeurwaerder et al., 2011	21910250	GSE20711	Fig. 2a-c	90
30	Sabatier et al., 2011b	20490655	GSE21653	Fig. 2a-c	240
31	Minn et al., 2005	16049480	GSE2603	Fig. 2a-c	99
32	Filipits et al., 2011	21807638	GSE26971	Fig. 2a-c	276
33	Sotiriou et al., 2006	16478745	GSE2990	Fig. 2a-c	102
34	Sabatier et al., 2011a	22110708	GSE31448	Fig. 2a-c	71
35	Rody et al., 2011	21978456	GSE31519	Fig. 2a-c	67
36	Miyake et al., 2012	22320227	GSE32646	Fig. 2a-c	115
37	Guo et al., 2013	24342436	GSE3494	Fig. 2a-c	251
38	Liu et al., 2012	22460789	GSE37946	Fig. 2a-c	41
39	Horak et al., 2013	23340299	GSE41998	Fig. 2a-c	279
40	Clarke et al., 2013	23740839	GSE42568	Fig. 2a-c	121

Continued on next page.

Study no.	Dataset reference	PubMed ID	Dataset ID/ availability [a]	Used for analysis in	Number of samples
41	Nagalla et al., 2013	23618380	GSE45255	Fig. 2a-c	139
42	Karn et al., 2010	19455418	GSE4611	Fig. 2a-c	153
43	Minn et al., 2007	17420468	GSE5327	Fig. 2a-c	58
44	Loi et al., 2007	17401012	GSE6532	Fig. 2a-c	82
45	Desmedt et al., 2007	17545524	GSE7390	Fig. 2a-c	198
46	Loi et al., 2008	18498629	GSE9195	Fig. 2a-c	77
47	Lefebvre et al., 2016	28027327	EGAS00001001695	Fig. 3a	213
48	Nik-Zainal et al., 2016	27135926	EGAS00001001178	Fig. 3a	569
49	Forbes et al., 2017	27899578	http://cancer.sanger.ac.uk/cosmic	Fig. 3a	2,446
50	Anders et al., 2008	18167534	GSE7849	Table 1	75
51	Buffa et al., 2011	21737487	GSE22219	Table 1	216
52	Chanrion et al., 2008	18347175	GSE9893	Table 1	155
53	Chin et al., 2006	17157792	E-TABM-158	Table 1	112
54	Chin et al., 2007	17925008	GSE8757	Table 1	171
55	Desmedt et al., 2007	17545524	GSE7390	Table 1	198
56	Filipits et al., 2011	21807638	GSE26971	Table 1	258
57	Hatzis et al., 2011	21558518	GSE25055	Table 1	309
58	Jezequel et al., 2009	19020972	GSE11264	Table 1	252
59	Kao et al., 2011	21501481	GSE20685	Table 1	296
60	Kuo et al., 2012	23049873	GSE33926	Table 1	51
61	Li et al., 2010	20098429	GSE19615	Table 1	115
62	Loi et al., 2007	17401012	GSE6532	Table 1	393
63	Loi et al., 2008	18498629	GSE9195	Table 1	77
64	Minn et al., 2005	16049480	GSE2603	Table 1	82
65	Minn et al., 2007	17420468	GSE5327	Table 1	58
66	Nagalla et al., 2013	23618380	GSE45255	Table 1	41
67	Pawitan et al., 2005	16280042	GSE1456	Table 1	159
68	Schmidt et al., 2008	18593943	GSE11121	Table 1	200
69	Sircoulomb et al., 2010	20932292	GSE17907	Table 1	39
70	Sotiriou et al., 2003	12917485	http://www.pnas.org/content/100/18/10393	Table 1	99
71	Van de Vijver et al., 2002	12490681	http://ccb.nki.nl/data/	Table 1	295
72	Wang et al., 2005	15721472	GSE2034	Table 1	286
73	Weigelt et al., 2005	16230372	GSE2741	Table 1	50
74	Zhang et al., 2009	18821012	GSE12093	Table 1	136
75	Zhou et al., 2007	17407600	GSE7378	Table 1	54

[a] Datasets are available via Gene Expression Omnibus (GEO): https://www.ncbi.nlm.nih.gov/gds (IDs starting with "GSE"), via ArrayExpress: https://www.ebi.ac.uk/arrayexpress (IDs starting with "E-"), via European Genome-phenome Archive (EGA): https://ega-archive.org (IDs starting with EGA) or via listed websites.

Table S2. Clinicopathological features of the breast cancer patients analyzed by immunohistochemistry

Clinicopathological variable	Number of patients (%) [a]	Median (Range) [b]
Pathology		
Normal breast	7 (7)	
Ductal breast carcinoma	100 (93)	
Gender		
Male	0 (0)	
Female	107 (100)	
Age		
<48	62 (62)	46 (27-74)
48-54	25 (25)	
>54	20 (19)	
Grade		
2	54 (56)	
3	42 (44)	
Tumor invasion		
T1	10 (10)	
T2	62 (62)	
T3	16 (16)	
T4	12 (12)	
Nodal status		
N0	59 (59)	
N1	31 (31)	
N2	10 (10)	
Metastasis		
M0	99 (99)	
M1	1 (1)	
Estrogen receptor status		
ER+	56 (57)	
ER-	42 (43)	
Progesterone receptor status		
PR+	35 (36)	
PR-	63 (64)	
HER2 status		
HER2+	36 (37)	
HER2-	62 (63)	

[a] Percentages shown under pathology and gender are with respect to all samples combined, including 100 tumor samples and 7 normal samples, while all other percentages are with respect to tumor samples only.

[b] Median age and range shown refer to patients only. We used 7 normal breast tissue samples from individuals with median age 41 and range 35-56.

Supplementary References

Anders, C.K., Acharya, C.R., Hsu, D.S., Broadwater, G., Garman, K., Foekens, J.A., Zhang, Y., Wang, Y., Marcom, K., Marks, J.R., *et al.* (2008). Age-specific differences in oncogenic pathway deregulation seen in human breast tumors. PLoS One *3*, e1373.

Bertheau, P., Turpin, E., Rickman, D.S., Espie, M., de Reynies, A., Feugeas, J.P., Plassa, L.F., Soliman, H., Varna, M., de Roquancourt, A., *et al.* (2007). Exquisite sensitivity of TP53 mutant and basal breast cancers to a dose-dense epirubicin-cyclophosphamide regimen. PLoS Med *4*, e90.

Bos, P.D., Zhang, X.H., Nadal, C., Shu, W., Gomis, R.R., Nguyen, D.X., Minn, A.J., van de Vijver, M.J., Gerald, W.L., Foekens, J.A., *et al.* (2009). Genes that mediate breast cancer metastasis to the brain. Nature *459*, 1005-1009.

Buffa, F.M., Camps, C., Winchester, L., Snell, C.E., Gee, H.E., Sheldon, H., Taylor, M., Harris, A.L., and Ragoussis, J. (2011). MicroRNA-associated progression pathways and potential therapeutic targets identified by integrated mRNA and microRNA expression profiling in breast cancer. Cancer Res *71*, 5635-5645.

Cancer Genome Atlas, N. (2012). Comprehensive molecular portraits of human breast tumours. Nature *490*, 61-70.

Chanrion, M., Negre, V., Fontaine, H., Salvetat, N., Bibeau, F., Mac Grogan, G., Mauriac, L., Katsaros, D., Molina, F., Theillet, C., *et al.* (2008). A gene expression signature that can predict the recurrence of tamoxifen-treated primary breast cancer. Clin Cancer Res *14*, 1744-1752.

Chin, K., DeVries, S., Fridlyand, J., Spellman, P.T., Roydasgupta, R., Kuo, W.L., Lapuk, A., Neve, R.M., Qian, Z., Ryder, T., *et al.* (2006). Genomic and transcriptional aberrations linked to breast cancer pathophysiologies. Cancer Cell *10*, 529-541.

Chin, S.F., Teschendorff, A.E., Marioni, J.C., Wang, Y., Barbosa-Morais, N.L., Thorne, N.P., Costa, J.L., Pinder, S.E., van de Wiel, M.A., Green, A.R., *et al.* (2007). High-resolution aCGH and expression profiling identifies a novel genomic subtype of ER negative breast cancer. Genome Biol *8*, R215.

Clarke, C., Madden, S.F., Doolan, P., Aherne, S.T., Joyce, H., O'Driscoll, L., Gallagher, W.M., Hennessy, B.T., Moriarty, M., Crown, J., *et al.* (2013). Correlating transcriptional networks to breast cancer survival: a large-scale coexpression analysis. Carcinogenesis *34*, 2300-2308.

Curtis, C., Shah, S.P., Chin, S.F., Turashvili, G., Rueda, O.M., Dunning, M.J., Speed, D., Lynch, A.G., Samarajiwa, S., Yuan, Y., *et al.* (2012). The genomic and transcriptomic architecture of 2,000 breast tumours reveals novel subgroups. Nature *486*, 346-352.

Dedeurwaerder, S., Desmedt, C., Calonne, E., Singhal, S.K., Haibe-Kains, B., Defrance, M., Michiels, S., Volkmar, M., Deplus, R., Luciani, J., *et al.* (2011). DNA methylation profiling reveals a predominant immune component in breast cancers. EMBO Mol Med *3*, 726-741.

Desmedt, C., Di Leo, A., de Azambuja, E., Larsimont, D., Haibe-Kains, B., Selleslags, J., Delaloge, S., Duhem, C., Kains, J.P., Carly, B., *et al.* (2011). Multifactorial approach to predicting resistance to anthracyclines. J Clin Oncol *29*, 1578-1586.

Desmedt, C., Giobbie-Hurder, A., Neven, P., Paridaens, R., Christiaens, M.R., Smeets, A., Lallemand, F., Haibe-Kains, B., Viale, G., Gelber, R.D., *et al.* (2009). The Gene expression Grade Index: a potential predictor of relapse for endocrine-treated breast cancer patients in the BIG 1-98 trial. BMC Med Genomics *2*, 40.

Desmedt, C., Piette, F., Loi, S., Wang, Y., Lallemand, F., Haibe-Kains, B., Viale, G., Delorenzi, M., Zhang, Y., d'Assignies, M.S., *et al.* (2007). Strong time dependence of the 76-gene prognostic signature for node-negative breast cancer patients in the TRANSBIG multicenter independent validation series. Clin Cancer Res *13*, 3207-3214.

Filipits, M., Rudas, M., Jakesz, R., Dubsky, P., Fitzal, F., Singer, C.F., Dietze, O., Greil, R., Jelen, A., Sevelda, P., *et al.* (2011). A new molecular predictor of distant recurrence in ER-positive, HER2-negative breast cancer adds independent information to conventional clinical risk factors. Clin Cancer Res *17*, 6012-6020.

Finak, G., Bertos, N., Pepin, F., Sadekova, S., Souleimanova, M., Zhao, H., Chen, H., Omeroglu, G., Meterissian, S., Omeroglu, A., *et al.* (2008). Stromal gene expression predicts clinical outcome in breast cancer. Nat Med *14*, 518-527.

Forbes, S.A., Beare, D., Boutselakis, H., Bamford, S., Bindal, N., Tate, J., Cole, C.G., Ward, S., Dawson, E., Ponting, L., *et al.* (2017). COSMIC: somatic cancer genetics at high-resolution. Nucleic Acids Res *45*, D777-D783.

Gluck, S., Ross, J.S., Royce, M., McKenna, E.F., Jr., Perou, C.M., Avisar, E., and Wu, L. (2012). TP53 genomics predict higher clinical and pathologic tumor response in operable early-stage breast cancer treated with docetaxel-capecitabine +/- trastuzumab. Breast Cancer Res Treat *132*, 781-791.

Guedj, M., Marisa, L., de Reynies, A., Orsetti, B., Schiappa, R., Bibeau, F., MacGrogan, G., Lerebours, F., Finetti, P., Longy, M., *et al.* (2012). A refined molecular taxonomy of breast cancer. Oncogene *31*, 1196-1206.

Guo, X., Zhu, S.X., Brunner, A.L., van de Rijn, M., and West, R.B. (2013). Next generation sequencing-based expression profiling identifies signatures from benign stromal proliferations that define stromal components of breast cancer. Breast cancer research : BCR *15*, R117.

Hatzis, C., Pusztai, L., Valero, V., Booser, D.J., Esserman, L., Lluch, A., Vidaurre, T., Holmes, F., Souchon, E., Wang, H., *et al.* (2011). A genomic predictor of response and survival following taxane-anthracycline chemotherapy for invasive breast cancer. Jama *305*, 1873-1881.

Horak, C.E., Pusztai, L., Xing, G., Trifan, O.C., Saura, C., Tseng, L.M., Chan, S., Welcher, R., and Liu, D. (2013). Biomarker analysis of neoadjuvant doxorubicin/cyclophosphamide followed by ixabepilone or Paclitaxel in early-stage breast cancer. Clin Cancer Res *19*, 1587-1595.

Jezequel, P., Campone, M., Roche, H., Gouraud, W., Charbonnel, C., Ricolleau, G., Magrangeas, F., Minvielle, S., Geneve, J., Martin, A.L., *et al.* (2009). A 38-gene expression signature to predict metastasis risk in node-positive breast cancer after systemic adjuvant chemotherapy: a genomic substudy of PACS01 clinical trial. Breast Cancer Res Treat *116*, 509-520.

Kao, K.J., Chang, K.M., Hsu, H.C., and Huang, A.T. (2011). Correlation of microarray-based breast cancer molecular subtypes and clinical outcomes: implications for treatment optimization. BMC Cancer *11*, 143.

Karn, T., Metzler, D., Ruckhaberle, E., Hanker, L., Gatje, R., Solbach, C., Ahr, A., Schmidt, M., Holtrich, U., Kaufmann, M., *et al.* (2010). Data-driven derivation of cutoffs from a pool of 3,030 Affymetrix arrays to stratify distinct clinical types of breast cancer. Breast Cancer Res Treat *120*, 567-579.

Karnoub, A.E., Dash, A.B., Vo, A.P., Sullivan, A., Brooks, M.W., Bell, G.W., Richardson, A.L., Polyak, K., Tubo, R., and Weinberg, R.A. (2007). Mesenchymal stem cells within tumour stroma promote breast cancer metastasis. Nature *449*, 557-563.

Korde, L.A., Lusa, L., McShane, L., Lebowitz, P.F., Lukes, L., Camphausen, K., Parker, J.S., Swain, S.M., Hunter, K., and Zujewski, J.A. (2010). Gene expression pathway analysis to predict response to neoadjuvant docetaxel and capecitabine for breast cancer. Breast Cancer Res Treat *119*, 685-699.

Kuo, W.H., Chang, Y.Y., Lai, L.C., Tsai, M.H., Hsiao, C.K., Chang, K.J., and Chuang, E.Y. (2012). Molecular characteristics and metastasis predictor genes of triple-negative breast cancer: a clinical study of triple-negative breast carcinomas. PLoS One *7*, e45831.

Lefebvre, C., Bachelot, T., Filleron, T., Pedrero, M., Campone, M., Soria, J.C., Massard, C., Levy, C., Arnedos, M., Lacroix-Triki, M., *et al.* (2016). Mutational Profile of Metastatic Breast Cancers: A Retrospective Analysis. PLoS Med *13*, e1002201.

Li, Y., Zou, L., Li, Q., Haibe-Kains, B., Tian, R., Li, Y., Desmedt, C., Sotiriou, C., Szallasi, Z., Iglehart, J.D., *et al.* (2010). Amplification of LAPTM4B and YWHAZ contributes to chemotherapy resistance and recurrence of breast cancer. Nat Med *16*, 214-218.

Liu, J.C., Voisin, V., Bader, G.D., Deng, T., Pusztai, L., Symmans, W.F., Esteva, F.J., Egan, S.E., and Zacksenhaus, E. (2012). Seventeen-gene signature from enriched Her2/Neu mammary tumor-initiating cells predicts clinical outcome for human HER2+:ERalpha- breast cancer. Proc Natl Acad Sci U S A *109*, 5832-5837.

Loi, S., Haibe-Kains, B., Desmedt, C., Lallemand, F., Tutt, A.M., Gillet, C., Ellis, P., Harris, A., Bergh, J., Foekens, J.A., *et al.* (2007). Definition of clinically distinct molecular subtypes in estrogen receptor-positive breast carcinomas through genomic grade. J Clin Oncol *25*, 1239-1246.

Loi, S., Haibe-Kains, B., Desmedt, C., Wirapati, P., Lallemand, F., Tutt, A.M., Gillet, C., Ellis, P., Ryder, K., Reid, J.F., *et al.* (2008). Predicting prognosis using molecular profiling in estrogen receptor-positive breast cancer treated with tamoxifen. BMC genomics *9*, 239.

Ma, X.J., Dahiya, S., Richardson, E., Erlander, M., and Sgroi, D.C. (2009). Gene expression profiling of the tumor microenvironment during breast cancer progression. Breast cancer research : BCR *11*, R7.

Minn, A.J., Gupta, G.P., Padua, D., Bos, P., Nguyen, D.X., Nuyten, D., Kreike, B., Zhang, Y., Wang, Y., Ishwaran, H., *et al.* (2007). Lung metastasis genes couple breast tumor size and metastatic spread. Proc Natl Acad Sci U S A *104*, 6740-6745.

Minn, A.J., Gupta, G.P., Siegel, P.M., Bos, P.D., Shu, W., Giri, D.D., Viale, A., Olshen, A.B., Gerald, W.L., and Massague, J. (2005). Genes that mediate breast cancer metastasis to lung. Nature *436*, 518-524.

Miyake, T., Nakayama, T., Naoi, Y., Yamamoto, N., Otani, Y., Kim, S.J., Shimazu, K., Shimomura, A., Maruyama, N., Tamaki, Y., *et al.* (2012). GSTP1 expression predicts poor pathological complete response to neoadjuvant chemotherapy in ER-negative breast cancer. Cancer Sci *103*, 913-920.

Nagalla, S., Chou, J.W., Willingham, M.C., Ruiz, J., Vaughn, J.P., Dubey, P., Lash, T.L., Hamilton-Dutoit, S.J., Bergh, J., Sotiriou, C., *et al.* (2013). Interactions between immunity, proliferation and molecular subtype in breast cancer prognosis. Genome Biol *14*, R34.

Nik-Zainal, S., Davies, H., Staaf, J., Ramakrishna, M., Glodzik, D., Zou, X., Martincorena, I., Alexandrov, L.B., Martin, S., Wedge, D.C., *et al.* (2016). Landscape of somatic mutations in 560 breast cancer whole-genome sequences. Nature *534*, 47-54.

Osmanbeyoglu, H. U., Pelossof, R., Bromberg, J. F., and Leslie, C. S. (2014). Linking signaling pathways to transcriptional programs in breast cancer. Genome Res *24*, 1869-1880.

Pawitan, Y., Bjohle, J., Amler, L., Borg, A.L., Egyhazi, S., Hall, P., Han, X., Holmberg, L., Huang, F., Klaar, S., *et al.* (2005). Gene expression profiling spares early breast cancer patients from adjuvant therapy: derived and validated in two population-based cohorts. Breast cancer research : BCR *7*, R953-964.

Perou, C.M., Sorlie, T., Eisen, M.B., van de Rijn, M., Jeffrey, S.S., Rees, C.A., Pollack, J.R., Ross, D.T., Johnsen, H., Akslen, L.A., *et al.* (2000). Molecular portraits of human breast tumours. Nature *406*, 747-752.

Popovici, V., Chen, W., Gallas, B.G., Hatzis, C., Shi, W., Samuelson, F.W., Nikolsky, Y., Tsyganova, M., Ishkin, A., Nikolskaya, T., *et al.* (2010). Effect of training-sample size and classification difficulty on the accuracy of genomic predictors. Breast cancer research : BCR *12*, R5.

Radvanyi, L., Singh-Sandhu, D., Gallichan, S., Lovitt, C., Pedyczak, A., Mallo, G., Gish, K., Kwok, K., Hanna, W., Zubovits, J., *et al.* (2005). The gene associated with trichorhinophalangeal syndrome in humans is overexpressed in breast cancer. Proc Natl Acad Sci U S A *102*, 11005-11010.

Richardson, A.L., Wang, Z.C., De Nicolo, A., Lu, X., Brown, M., Miron, A., Liao, X., Iglehart, J.D., Livingston, D.M., and Ganesan, S. (2006). X chromosomal abnormalities in basal-like human breast cancer. Cancer Cell *9*, 121-132.

Rody, A., Karn, T., Liedtke, C., Pusztai, L., Ruckhaeberle, E., Hanker, L., Gaetje, R., Solbach, C., Ahr, A., Metzler, D., *et al.* (2011). A clinically relevant gene signature in triple negative and basal-like breast cancer. Breast cancer research : BCR *13*, R97.

Sabatier, R., Finetti, P., Adelaide, J., Guille, A., Borg, J.P., Chaffanet, M., Lane, L., Birnbaum, D., and Bertucci, F. (2011a). Down-regulation of ECRG4, a candidate tumor suppressor gene, in human breast cancer. PLoS One *6*, e27656.

Sabatier, R., Finetti, P., Cervera, N., Lambaudie, E., Esterni, B., Mamessier, E., Tallet, A., Chabannon, C., Extra, J.M., Jacquemier, J., *et al.* (2011b). A gene expression signature identifies two prognostic subgroups of basal breast cancer. Breast Cancer Res Treat *126*, 407-420.

Schmidt, M., Bohm, D., von Torne, C., Steiner, E., Puhl, A., Pilch, H., Lehr, H.A., Hengstler, J.G., Kolbl, H., and Gehrmann, M. (2008). The humoral immune system has a key prognostic impact in node-negative breast cancer. Cancer Res *68*, 5405-5413.

Shi, L., Campbell, G., Jones, W.D., Campagne, F., Wen, Z., Walker, S.J., Su, Z., Chu, T.M., Goodsaid, F.M., Pusztai, L., *et al.* (2010). The MicroArray Quality Control (MAQC)-II study of common practices for the development and validation of microarray-based predictive models. Nat Biotechnol *28*, 827-838.

Sircoulomb, F., Bekhouche, I., Finetti, P., Adelaide, J., Ben Hamida, A., Bonansea, J., Raynaud, S., Innocenti, C., Charafe-Jauffret, E., Tarpin, C., *et al.* (2010). Genome profiling of ERBB2-amplified breast cancers. BMC Cancer *10*, 539.

Sotiriou, C., Neo, S.Y., McShane, L.M., Korn, E.L., Long, P.M., Jazaeri, A., Martiat, P., Fox, S.B., Harris, A.L., and Liu, E.T. (2003). Breast cancer classification and prognosis based on gene expression profiles from a population-based study. Proc Natl Acad Sci U S A *100*, 10393-10398.

Sotiriou, C., Wirapati, P., Loi, S., Harris, A., Fox, S., Smeds, J., Nordgren, H., Farmer, P., Praz, V., Haibe-Kains, B., *et al.* (2006). Gene expression profiling in breast cancer: understanding the molecular basis of histologic grade to improve prognosis. J Natl Cancer Inst *98*, 262-272.

Symmans, W.F., Hatzis, C., Sotiriou, C., Andre, F., Peintinger, F., Regitnig, P., Daxenbichler, G., Desmedt, C., Domont, J., Marth, C., *et al.* (2010). Genomic index of sensitivity to endocrine therapy for breast cancer. J Clin Oncol *28*, 4111-4119.

Tabchy, A., Valero, V., Vidaurre, T., Lluch, A., Gomez, H., Martin, M., Qi, Y., Barajas-Figueroa, L.J., Souchon, E., Coutant, C., *et al.* (2010). Evaluation of a 30-gene paclitaxel, fluorouracil, doxorubicin, and cyclophosphamide chemotherapy response predictor in a multicenter randomized trial in breast cancer. Clin Cancer Res *16*, 5351-5361.

Turashvili, G., Bouchal, J., Baumforth, K., Wei, W., Dziechciarkova, M., Ehrmann, J., Klein, J., Fridman, E., Skarda, J., Srovnal, J., *et al.* (2007). Novel markers for differentiation of lobular and ductal invasive breast carcinomas by laser microdissection and microarray analysis. BMC Cancer *7*, 55.

van de Vijver, M.J., He, Y.D., van't Veer, L.J., Dai, H., Hart, A.A., Voskuil, D.W., Schreiber, G.J., Peterse, J.L., Roberts, C., Marton, M.J., *et al.* (2002). A gene-expression signature as a predictor of survival in breast cancer. The New England journal of medicine *347*, 1999-2009.

Wang, Y., Klijn, J.G., Zhang, Y., Sieuwerts, A.M., Look, M.P., Yang, F., Talantov, D., Timmermans, M., Meijer-van Gelder, M.E., Yu, J., *et al.* (2005). Gene-expression profiles to predict distant metastasis of lymph-node-negative primary breast cancer. Lancet *365*, 671-679.

Weigelt, B., Hu, Z., He, X., Livasy, C., Carey, L.A., Ewend, M.G., Glas, A.M., Perou, C.M., and Van't Veer, L.J. (2005). Molecular portraits and 70-gene prognosis signature are preserved throughout the metastatic process of breast cancer. Cancer Res *65*, 9155-9158.

Zhang, Y., Sieuwerts, A.M., McGreevy, M., Casey, G., Cufer, T., Paradiso, A., Harbeck, N., Span, P.N., Hicks, D.G., Crowe, J., *et al.* (2009). The 76-gene signature defines high-risk patients that benefit from adjuvant tamoxifen therapy. Breast Cancer Res Treat *116*, 303-309.

Zhao, H., Langerod, A., Ji, Y., Nowels, K.W., Nesland, J.M., Tibshirani, R., Bukholm, I.K., Karesen, R., Botstein, D., Borresen-Dale, A.L., *et al.* (2004). Different gene expression patterns in invasive lobular and ductal carcinomas of the breast. Mol Biol Cell *15*, 2523-2536.

Zhou, Y., Yau, C., Gray, J.W., Chew, K., Dairkee, S.H., Moore, D.H., Eppenberger, U., Eppenberger-Castori, S., and Benz, C.C. (2007). Enhanced NF kappa B and AP-1 transcriptional activity associated with antiestrogen resistant breast cancer. BMC Cancer *7*, 59.
